# Desmoid Tumors: Current Perspective and Treatment

**DOI:** 10.1007/s11864-024-01177-5

**Published:** 2024-01-25

**Authors:** Ankit Mangla, Nikki Agarwal, Gary Schwartz

**Affiliations:** 1 University Hospitals Seidman Cancer Center, Cleveland, OH USA; 2 Case Comprehensive Cancer Center, Cleveland, OH USA; 3 Case Western Reserve University School of Medicine, 11100 Euclid Avenue, Lakeside Suite#1200, Room 1243, Cleveland, OH 44106 USA; 4 Cleveland Clinic Children’s Hospitals, Cleveland, OH USA

**Keywords:** Desmoid tumors, Gamma-secretase, Sorafenib, Nirogacestat, Wnt pathway

## Abstract

Desmoid tumors are rare tumors with a tendency to infiltrate locally. The lack of a standard treatment approach makes choosing the most appropriate treatment for patients challenging. Most experts recommend watchful observation for asymptomatic patients as spontaneous regression of tumor is observed in up to 20% of patients. Upfront resection of the desmoid tumor has fallen out of favor due to high morbidity and high relapse rates associated with the tumor. Systemic therapy has evolved over several decades. Where chemotherapy, hormonal therapy, and non-steroidal anti-inflammatory drugs were used over the last several decades, tyrosine kinase inhibitors came to the forefront within the last decade. Most recently, gamma-secretase inhibitors have shown significant clinical benefit in patients with desmoid tumors, bringing forth an entirely new mechanistic approach. Several Wnt pathway inhibitors are also under development. Invasive approaches like cryoablation have also shown clinical benefit in patients with extra-abdominal desmoid tumors in recent years. The recent approval of nirogacestat has ushered in a new era of treatment for patients diagnosed with desmoid tumors. Several new molecules are expected to be approved over the coming years.

## Introduction

Desmoid tumor (DT) is a rare tumor characterized by clonal fibroblastic proliferation with a tendency to infiltrate locally [1]. DTs never metastasize. However, multifocal occurrence of DT has been reported [2, 3]. There is no standard treatment approach to these unpredictable tumors that can undergo spontaneous regression in up to 20% of patients. Surgical resection as the first line of treatment has fallen out of favor due to significant morbidity and high recurrence rates [4]. Medical therapy includes anthracycline-based chemotherapy, vinca-alkaloid-based chemotherapy, and targeted therapies, including tyrosine kinase inhibitors (TKI). The combination of non-steroidal anti-inflammatory drugs (NSAIDs) and hormone therapy is discouraged due to the lack of a clear benefit in prospective studies [5•, 6]. Newer drugs like gamma-secretase inhibitors have shown promise and are currently pending approval from the USFDA (United States Food and Drug Administration). Cryoablation is a relatively newer technique that is currently under investigation for the treatment of patients with DT. Several new molecules are currently being developed to target the Wnt pathway. This review will discuss the newer treatment approaches to this rare, yet significantly morbid disease.

## Epidemiology

DT is a rare cancer with a global incidence of two to six new diagnoses per million of the population per year [7–10]. In the USA, an estimated 1000 new patients are diagnosed with DT yearly [9]. More than 90% of cases of DT are sporadic and associated with a mutation in the β-catenin gene (*CTNNB1*). A minority of DTs are diagnosed in patients with germline APC mutation, which manifests as familial adenomatous polyposis (FAP) [11, 12]. The incidence of DT in patients with FAP is estimated at 3–30% [12]. However, approximately 8% of patients with sporadic DT are reported in patients with a family history of colon cancer, suggesting a genetic predisposition to the disease [13]. Most patients are diagnosed between the ages of 15 and 60 years; however, the peak incidence is seen between 30 and 40 years of age. DT affects women at a disproportionately higher rate compared to men [14].

## Clinical presentation and diagnosis

Patients with DT can be asymptomatic at the time of presentation. The symptoms arise due to pain and deformity caused by compression of local organs or neuro-vascular structures or erosion and destruction of adjacent musculoskeletal structures. The clinical course is unpredictable, where up to 20% of patients may experience spontaneous regression. The diagnosis is established with a core needle biopsy demonstrating long sweeping fascicles of bland fibroblasts/myofibroblasts. However, several morphologic patterns have been reported, including conventional, hyalinized/hypocellular, staghorn vessel, myxoid, keloidal, nodular fasciitis-like, and hypercellular patterns [15]. The imaging for DT relies mainly on CT (computed tomography) scans and MRI (magnetic resonance imaging) [16]. MRI is the most suitable modality for extra-abdominal DT as it allows an accurate description of the tumor and its relation with surrounding structures. ^18^F-FDG-PET (Fludeoxyglucose F18- positron emission tomography) is not suitable for the evaluation of DT, as the median maximum standardized uptake is 4.1 (range from 1.0 to 8.1) [17].

## Pathogenesis

**Fig. 1:**
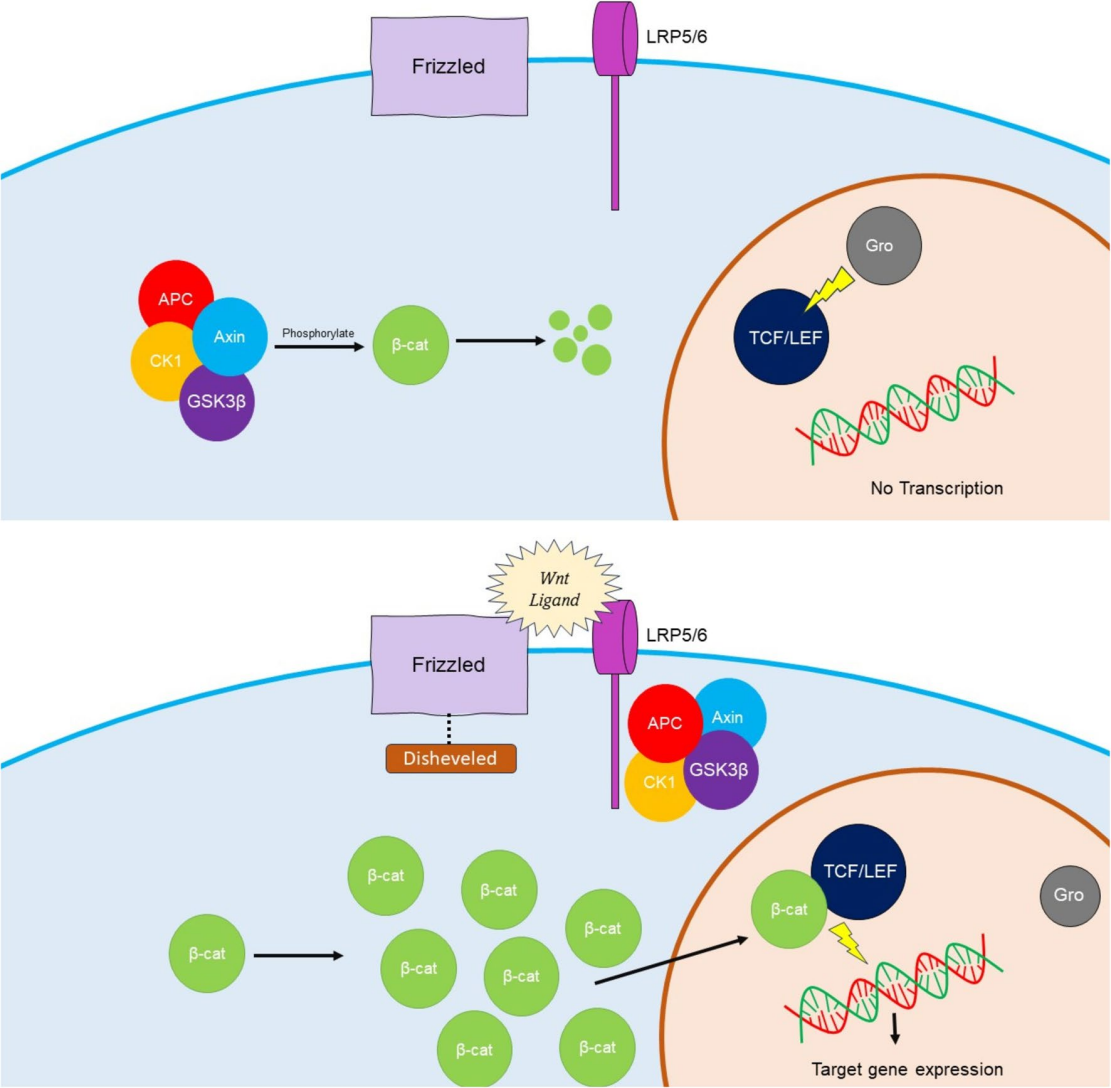
Wnt/ β-catenin signaling pathway: *Top panel*: In the absence of Wnt ligands, β-catenin is destroyed in the cytoplasm by the destruction complex comprising of APC, Axin, CK1, and GSK3β, preventing its translocation to the nucleus. In the absence of intra-nuclear β-catenin, TCF/LEF (family of transcription factors) interacts with Gro and represses transcription. *Bottom panel:* In the presence of the Wnt ligand, the destruction complex (comprising APC, Axin, CK1, and GSK3β) gets inactivated by binding with LRP5/6. This allows β-catenin to accumulate in the cytoplasm and translocate to the nucleus. In the nucleus, β-catenin dislodges Gro from TCF/LEF and activates the expression of genes. (APC, Adenomatous polyposis coli; CK1, Casein Kinase 1; Gro, Groucho; GSK3β, Glycogen Synthase Kinase 3β; LRP 5/6, Low-Density Lipoprotein receptor-related proteins 5 and 6; TCF/LEF, T-cell/Lymphoid Enhancer Factor; β-cat, β-catenin).

The dysregulation of the Wnt (Wingless/Integrated) pathway is noted in the development of several cancers [18]. In DT, constitutive activation caused by mutations in the β-catenin oncogene CTNNB1 (in sporadic cases) [19], or germline activation of the APC (adenomatous polyposis coli) gene (in patients with FAP) [20], activates the Wnt pathway, which prevents degradation of the cytosolic β-catenin. The cytosolic β-catenin protein translocates to the nucleus. It binds with the T-cell factor/lymphoid enhancer factor (TCF/LEF) family of transcription factors, displaces Groucho (Gro), and activates expression of target Wnt genes [21, 22]. Some of these genes are involved in proliferation and fibrosis (ADAM12, Fap-1α, WISP1, and SOX11). In contrast, others are involved in angiogenesis (VEGF—vascular endothelial growth factor) and activation of growth factor receptors (COX2 (Cyclo-oxygenase-2) activating platelet-derived growth factor (PDGF)-α and β) [21–23] (Fig. 1).

**Fig. 2:**
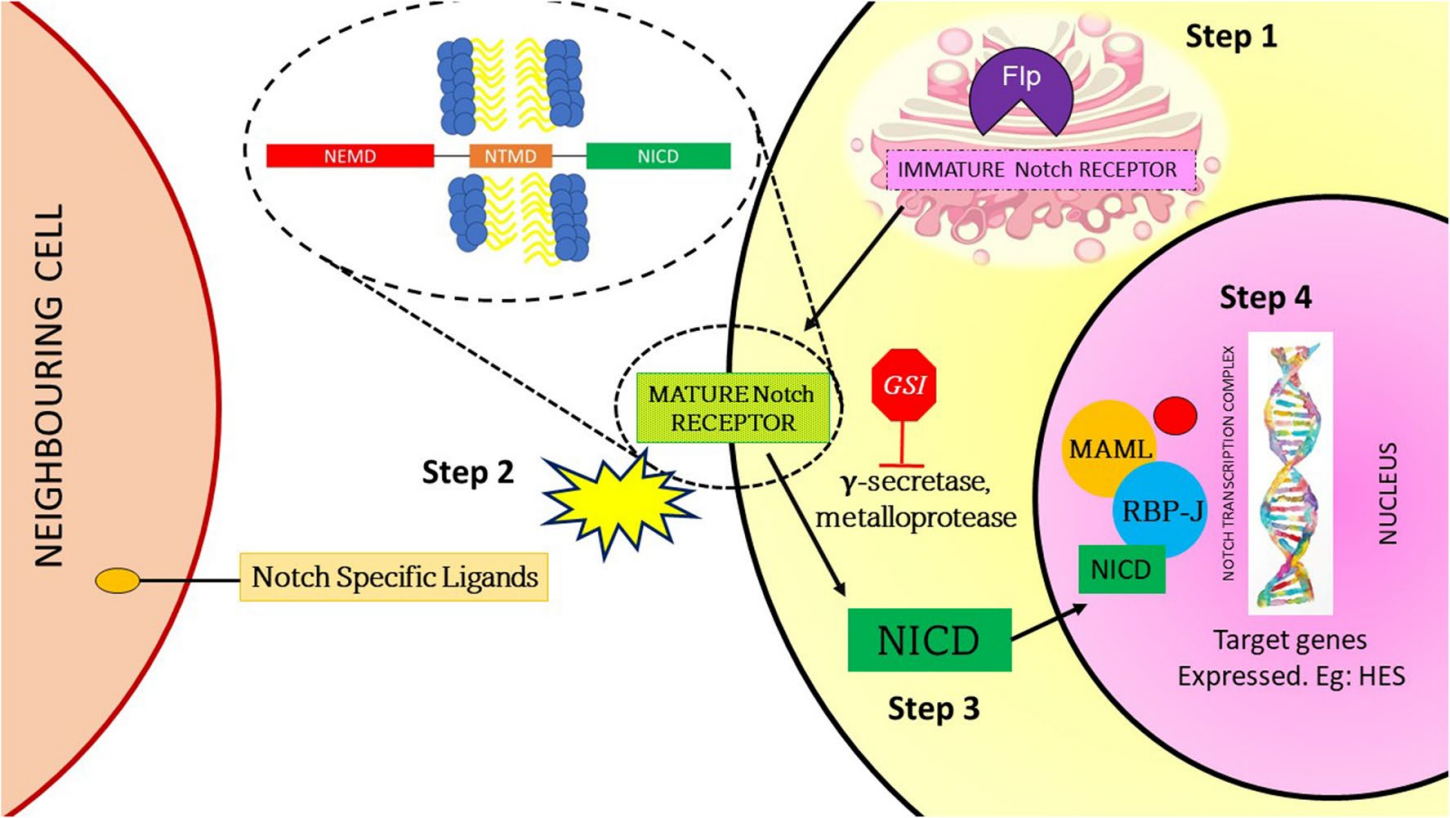
Notch Signaling Pathway: Step 1 (occurs in the cytosol): Furin-like protease (Flp) cleaves the immature Notch receptor in the Golgi apparatus. Step 2: Mature notch receptor has an extracellular, transmembrane, and intracellular domain. The extracellular domain interacts with Notch-specific ligands (Jagged-1/2 or Delta-like ligand-1/3/4). Step 3: Mature Notch receptor is cleaved by metalloproteases (ADAM 10/17) and γ-secretase, releasing the Notch-intracellular domain. Step 4: Notch-intracellular domain translocates to the nucleus and forms a complex with transcription factors recombination signal binding protein for immunoglobulin kappa J region (RBP-J) and Mastermind-like proteins (MAML), which leads to transcription of proteins. (Flp, Furin-like protein; GSI, Gamma-Secretase Inhibitor; HES, Hairy Enhancer of Split; MAML, Mastermind-like proteins; NEMD, Notch-Extracellular Domain; NICD, Notch-Intracellular Domain; NTMD, Notch-Transmembrane Domain; RBP-J, Recombination Signal Binding Protein for Immunoglobulin Kappa J region).

Although the Wnt pathway is directly implicated in the pathogenesis of DT, the crosstalk with the Notch signaling pathway is critical [24–26], as druggable targets, like γ-secretase inhibitors (GSI), are targeting the substrates in the Notch signaling pathway [27]. All homologous Notch receptors are transmembrane proteins that play a critical role in cell formation, differentiation, and apoptosis [28]. Dysregulation of the Notch signaling pathway is also implicated in the development of lymphoid leukemia and several solid tumor malignancies [29]. The interaction between the Notch signaling pathway and the Wnt pathway has been demonstrated in cultured cancer cells, and several animal models [25, 26]. In patients with FAP, β-catenin-mediated upregulation of Notch-specific ligand Jagged-1/2 (JAG-1) has been shown to activate the Notch signaling [24]. Several studies on DT tissues and cell strains have also demonstrated increased expression of the Notch-related gene HES1 (Hairy Enhancer of Split), [27] confirming that dysregulation of the Wnt pathway affects the Notch signaling pathway (Fig. 2).

## Wnt inhibitors

Since the discovery of the Wnt-1 gene in 1982, several advances have been made in understanding the role of the Wnt/β-catenin pathway [30]. The direct implications of dysregulation in the Wnt pathway causing tumorigenesis make it an attractive target for drug development [31]. However, targeting the Wnt pathway is challenging because of its intricate involvement in embryogenesis and adult tissue homeostasis [31]. Several drugs are in development targeting various ligands in the Wnt/β-catenin pathway.

Tegavivint (formerly BC2059) is a first-in-class, small molecule that inhibits the interaction between the β-catenin and transducing β-like protein-1 (TBL1) and its related protein (TBLR1). The absence of TBL1-TBLR1 significantly reduces the recruitment of the β-catenin to the Wnt target-gene promoters (*Axin* and *c-Myc*), resulting in the downregulation of transcription of target genes [32]. Also, the absence of TBL1/TBLR1 in the knockout cells (HT29 cells) leads to spontaneous apoptosis, consistent with the anti-apoptotic function of the Wnt/β-catenin pathway [32]. The results of the phase 1/2 study in adults and the pediatric age group were presented recently [33•, 34•]. In adults, 24 patients were enrolled in the phase 1 dose escalation and expansion study. The recommended phase 2 dose (RP2D) was declared 5 mg/kg intravenously. The median half-life was 38 h, supporting once-weekly administration. Treatment-related AE (TRAE) occurred in 20% of patients (fatigue, headache, nausea, constipation, dysgeusia, and decreased appetite). Only one patient each had grade 3 headache, diarrhea, increased alanine transaminase, hypophosphatemia, and stomatitis. No grade 4 or 5 TRAE were recorded. The overall objective response rate (ORR) was 17%. At RP2D, the ORR was 25% (two out of nine patients). However, significant tumor reduction was noted in these two patients (− 86% and − 91%). The 9-month progression-free survival (PFS) rate was 79% amongst those treated with RP2D [34•]. In the pediatric study, several tumor types, including five patients with DT, were enrolled in the phase 1 part of the study. The RP2D in children is determined at 6.5 mg/kg with no observed dose-limiting toxicity (DLT). The results from the dose expansion cohort are eagerly awaited [33•].

E7386 is a novel small molecule inhibiting the binding of β-catenin to its transcriptional coactivator CREB (cAMP-response element binding protein)-binding protein (CBP). Recently, updated results of the phase 1 dose escalation cohort reported a partial response in one patient with APC-mutated DT (NCT03833700). The RP2D was determined at 120 mg orally twice a day [35]. Ipafricept (formerly OMP-54F28) is a truncated frizzeld-8 receptor fused to the Immunoglobulin-G1 (IgG1) Fc region. It blocks the Wnt signaling by binding to the Wnt ligands. In the phase 1 study of ipafricept, two patients with desmoid tumors had stable disease for over 6 months, whereas one patient stayed on the drug for 98 months (the patient stopped the medicine out of his preference). [36]

## Gamma secretase inhibitors

The crosstalk between Wnt and Notch signaling pathways provides a scientific rationale for targeting γ-secretase. γ-secretase is a membrane-bound protease complex that cleaves the mature Notch receptor and releases the Notch intracellular domain (NICD). NICD translocates to the nucleus and facilitates transcription of HES, which is critical to the pathogenesis of DT. By inhibiting the activity of γ-secretase, the expression of critical Notch-related genes is prohibited (Fig. 2). Several GSIs are in development, amongst whom nirogacestat is currently in the most advanced stage and is pending approval by the USFDA.

## Nirogacestat

Nirogacestat, formerly called PF-03084014, is a selective, noncompetitive, reversible GSI that was recently studied in a double-blind, phase 3 randomized, placebo-controlled trial. The trial met its primary endpoint of PFS benefit. During the initial stages of clinical development, administration of nirogacestat demonstrated activity in patients with DT by inducing a partial response in five and prolonged disease stabilization in two out of seven patients [37, 38]. Downregulation of HES-4 was demonstrated in these patients, hence establishing the mechanistic rationale of administering a GSI in a patient with DT.

In phase 3, DeFi (Desmoid/Fibromatosis) trial, the risk of disease progression was 71% lower in the patients treated with nirogacestat than those treated with placebo (hazard ratio (HR)—0.29; 95% confidence interval (CI), 0.15 to 0.55; *P* < 0.001). The median PFS was not reached in the nirogacestat group at the end of the 15-month follow-up. The likelihood of being event free was higher with nirogacestat than with placebo at both the 1-year mark (85% versus 53%) and 2-year mark (76% versus 44%). In the subgroup analysis, the PFS benefit was seen regardless of gender, tumor location, previous treatments (or treatment naïve), and in those with FAP syndrome. Forty-one percent of patients had an objective response with nirogacestat compared to 8% with placebo (*P* < 0.001). Complete response was exclusively noted in 7% of patients receiving nirogacestat. The median time to first response was also much shorter with nirogacestat than placebo (5.6 months versus 11.1 months), and the median best percent change in the size of the tumor was − 27.1% with nirogacestat (+ 2.3% with placebo).

At the 15-month follow-up, the nirogacestat group also reported better patient-reported outcomes. Significantly better scores were recorded in the European Organization for the Research and Treatment of Cancer Quality of Life Questionnaire (EORTC QLQ)-C30 physical and role functioning scores, as well as quality of life scores. The GODDESS score (GOunder/Desmoid Tumor Research Foundation DEsmoid Symptom/Impact Scale) is an innovative tool for the assessment of patient-reported signs and symptoms that was used in the DeFi trial [39]. The BPI-SF (Brief Pain Inventory-Short Form) pain intensity score recorded a significantly better improvement in the average worst pain intensity with nirogacestat, indicating better patient outcomes with the GSI than with placebo. However, the clinicians must note that although these differences in patient-reported outcomes became statistically significant by cycle 10, the published results of the trial reflect a sharp decline in the pain intensity by cycle 2 (BPI-SF pain intensity score) and improvement in quality of life (reflected in the GODDESS score).

Nirogacestat also has several TRAE. Any grade TRAE was noted in all patients, and grade 3/4 TRAE was noted in 55% of patients. Diarrhea was the most common TRAE (84% of patients) amongst all TRAEs in the nirogacestat group, followed by nausea (54%), fatigue (51%), and hypophosphatemia (42%). The most interesting of all the TRAEs was ovarian dysfunction, which was reported in 75% of the patients in childbearing age (27 out of 36 subjects). Amenorrhea was the most common symptom reported within the milieu of ovarian dysfunction. Resolution of symptoms was noted in nine out of 14 subjects while they continued on the treatment. All subjects experienced complete resolution of the symptoms after stopping nirogacestat (for any reason) [40]. The ovarian dysfunction from nirogacestat is hypothesized to be secondary to disruption of the Notch pathway in the pre-antral follicles. Notch signaling is critical to ovarian follicle development [41]. However, it must be noted that no reports of “loss of reproductive potential” have been attributed to nirogacestat to date. It is partly due to the fact that “reproductive potential” is a complicated issue to assess, which also depends on the desire to get pregnant. It is also reassuring to know that the symptoms of ovarian dysfunction resolved in all subjects who discontinued nirogacestat. Until real-world experience or long-term follow-up studies are available, ovarian dysfunction from nirogacestat should not be interpreted as “loss of reproductive potential” and should not be considered a prohibitive factor to prescribe this drug to patients in the reproductive age group.

## AL 101 and AL-102

AL-101 (formerly BMS-906024) and AL102 (formerly BMS-986115) are other noteworthy GSIs under investigation. AL-101 is an intravenous formulation, and AL-102 is an oral formulation. Otherwise, the two compounds are structurally similar. In the phase 1 dose escalation study of AL-101, out of the three enrolled patients with DT, one patient achieved a partial response (PR), and one patient had stable disease (SD) [42]. Likewise, in the phase 1 dose escalation study of AL-102, one patient with DT was enrolled and had SD at 6 months (~ 16.5% reduction in tumor burden by response evaluation criteria in solid tumors (RECIST)).

The phase 2/3 RINGSIDE trial is evaluating the efficacy of AL-102. The phase 2 portion of the study looked at three different dosing regimens [43]. The patients in the 1.2 mg once daily (QD) dosing cohort achieved a quicker, deeper, and more sustained response than the other two cohorts (2 mg intermittent biweekly and 4 mg intermittent biweekly). Six out of 12 patients in the cohort (of 1.2 mg QD) had PR, and the other six had SD. The patients in this cohort had a significantly higher reduction in tumor burden (− 51.9%) and T2-weighted signal intensity (− 58.4%) by week 16. No grade 4 or 5 TRAEs were reported with AL-102. Ovarian dysfunction was reported in three out of nine patients (of reproductive age group) in the 1.2 mg QD cohort [44]. The phase 3 portion is currently active and recruiting (NCT04871282).

## Vascular endothelial growth factor inhibitors

The dysregulation in the Wnt pathway increases the expression of genes that transcribe VEGF and PDGF. VEGF and PDGF are pivotal in tumor-associated angiogenesis via autocrine and paracrine mechanisms [45]. Several TKIs targeting the growth factors have been shown to be of benefit to patients with DT.

## Sorafenib

Sorafenib is a multikinase inhibitor that suppresses tumor cell proliferation by inhibiting B-Raf, Raf-1, and kinase activity in the Ras/Raf/MEK/ERK signaling pathways. In addition, sorafenib also targets VEGF, PDGFR-β, c-KIT, and other proteins that promote tumor angiogenesis [46]. Anecdotal studies demonstrated the activity of sorafenib [47], which prompted a phase 3 randomized trial where subjects with aggressive DT (> 10% radiological increase in 6 months) were randomized in a 2:1 fashion between sorafenib (at 400 mg by mouth daily) and placebo in a blinded fashion [48•]. At progression, the patients were unblinded, and those receiving a placebo were allowed to crossover to the sorafenib arm.

Out of 87 patients enrolled in the trial, 50 patients received sorafenib, and 37 received a placebo. The PFS benefit was evident in the sorafenib group, with 87% lower risk of progression or death with sorafenib than placebo (HR for death—0.13; 95% CI, 0.05–0.31; *P* < 0.001) [48•]. The overall objective response was noted in 33% of patients (16 out of 49 patients) in the sorafenib group (one patient had a complete response (CR), and 15 patients had PR) and 20% of patients (seven out of 35, all PR) in the placebo group. The sorafenib group outperformed the placebo group in reducing the size of target lesions (− 26% versus − 12%) and achieved the RECIST-defined response quicker than placebo (9.6 months versus 13.3 months). No new safety signals were reported with sorafenib. Forty-seven percent of patients experienced a grade 3/4 TRAE with sorafenib, where skin rash (14%) was the most common TRAE, followed by hypertension (8%) [48•].

## Pazopanib

Pazopanib is another multikinase inhibitor that targets the VEGF receptor, PDFGR, and c-KIT, thus inhibiting angiogenesis [49]. The USFDA approved it as a second-line treatment in patients diagnosed with soft-tissue sarcoma. DESMOPAZ is a non-comparative, randomized, phase 2 trial examining the efficacy of pazopanib (800 mg by mouth daily) in patients with DT and methotrexate-vinblastine combination independently [50]. It must be noted that although no statistical comparison was planned between the two groups, the crossover was allowed in case of disease progression [50].

In the pazopanib group, the 1-year and 2-year PFS rates were 85.6% and 67.2%, respectively. The 1-year and 2-year PFS rate in the chemotherapy group was 79.0% at both timepoints. Pazopanib induced a PR in 37% (17 out of 46) patients, which was the best overall response. Twenty-seven out of 46 patients (58.7%) had stable disease. Diarrhea, hypertension, and fatigue were the most common TRAEs (any grade—81%, 80%, and 44%, respectively). Grade 3 (or higher) TRAEs in the form of diarrhea, hypertension, and fatigue were noted in 15%, 19%, and 6% of patients, respectively. No new safety signals were observed in this study [50].

## Imatinib

Imatinib is a selective tyrosine kinase inhibitor targeting the Abelson (ABL) tyrosine kinase, PDGFR, and KIT. Several single-arm, open-label, phase 2 trials of imatinib have been reported over the years, demonstrating an ORR between 6 and 20% [51–54]. In the largest phase 2 study from the Sarcoma Alliance for Research through Collaboration (SARC), exploring imatinib in patients with unresectable DT, fifty-one patients were enrolled across five institutions. Three patients achieved partial response by RECIST at 19, 22, and 26 months, and stable disease was reported in 84% of patients at the 4-month follow-up [51]. The 1-year and 2-year PFS rates were recorded at 66% and 58%, respectively. In this trial, the dose of imatinib was decided based on body surface area (BSA). Patients with a BSA of ≥ 1.5 m^2^ received 300 mg orally twice daily, BSA from 1.0 to 1.49 m^2^ received 200 mg orally twice daily, and BSA of < 1.0 m^2^ received 100 mg orally twice daily. Neutropenia, rash, and fatigue were reported as grade 3 events in more than 5% of patients. No new toxicity signals were identified [51].

## Sunitinib

Sunitinib is a multikinase inhibitor targeting VEGF-receptor-1, 2, and 3; PDGFR-α and β, KIT (CD117), RET, CSFR-1, and FLT-3 receptors [55]. In a phase two prospective trial from Seoul, Korea, nineteen patients with advanced, unresectable DT were recruited in a Simon-two-stage design [56]. Sunitinib was given in a monthly cycle, dosed at 37.5 mg daily for 4 weeks. At the 8-week mark, five patients had a partial response (26.3%), eight patients had stable disease (42.1%), and the overall disease control rate (DCR) was 68.4%. The 2-year PFS rate was 74.7%. Neutropenia was the most common grade 3 (or higher) TRAE [56]. In the phase 2 randomized trial of sunitinib at 52 mg orally daily versus tamoxifen and meloxicam, the ORR was 75% (17 out of 22 patients), and the 2-year PFS rate was 81%. The results of this trial are yet to be published [57].

## Cryoablation

Cryoablation is a percutaneous ablation technique where liquefied nitrogen or argon creates very low temperatures inside the tumor tissue. The cytotoxic activity starts at − 20 °C. Cryoprobe is inserted into the tumor tissue, and liquid nitrogen thaws as it flows through the probe, dropping the temperature to as low as − 190 °C. After the freezing phase, a thawing phase begins where a heating probe, or helium, is used to heat the tumor. The freezing and thawing process is repeated multiple times to achieve cytotoxic effects [58]. Cryoablation has been explored in desmoid tumors in multiple anecdotal and a few prospective studies [59–62].

CRYODESMO-1 is a prospective study of patients with extra-abdominal prospective DT. Only patients with progressive DT that were not amenable to resection were included in this study. The primary endpoint of this study was to observe the non-progression rate at 12 months. An impressive number of patients achieved CR (12 of 42 patients) and PR (11 of 42 patients), with an ORR of 55%. No progression at 12 months was noted in 36 of 42 patients (86%), thus meeting the primary endpoint of the trial. Median PFS was not reached on the 31-month long-term follow-up. The majority of patients (45 of 50) were treated in one procedure, whereas five patients needed a two-step procedure. Rhabdomyolysis was the most common grade 3/4 AE noted in patients undergoing cryoablation. A transient increase in pain after the procedure was noted for 1–2 days. However, the average pain score started declining from month 2, along with analgesic use. One distinct advantage of cryoablation over other techniques is visualization of the tumor during the procedure with the use of CT scans, which is usually not available with other modalities like radiofrequency ablation (RFA) [62].

## Conclusion

DT is a localized neoplasm with no potential to metastasize and an unpredictable course. While a “wait and watch” strategy is suitable for asymptomatic patients, systemic therapy is appropriate for symptomatic patients, especially those with Gardeners syndrome. Upfront surgical resection and hormonal therapy with or without NSAIDs are not recommended in the current treatment landscape. TKI (like sorafenib) and GSI (like nirogacestat) have shown significant benefits in randomized phase 3 trials, inducing response and improving the symptoms of patients with DT. Several other molecules targeting the gamma-secretase and Wnt pathway are currently being developed. Amongst localized therapies, cryoablation is an attractive option in patients with extra-abdominal DT. However, careful selection of patients is needed for such procedures.

## Data Availability

No datasets were generated or analysed during the current study.

## Abbreviations

^18^F-FDG-PET: Fludeoxyglucose F18-positron emission tomography
ABL: Abelson
APC: Adenomatous polyposis coli
BPI-SF: Brief Pain Inventory-Short Form
BSA: Body surface area
CBP: CREB-binding protein
CI: Confidence interval
COX: Cyclooxygenase
CR: Complete response
CREB: CAMP-response element binding protein
CT: Computed tomography
CTNNB1: β-Catenin gene
DCR: Disease control rate
DLT: Dose limiting toxicity
DT: Desmoid tumor
EORTC QLQ: European Organization for the Research and Treatment of Cancer Quality of Life Questionnaire
FAP: Familial adenomatous polyposis
GODDESS: GOunder/Desmoid Tumor Research Foundation DEsmoid Symptom/Impact Scale
Gro: Groucho
GSI: Gamma-secretase inhibitor
HES-1: Hairy Enhancer of Split
HR: Hazard ratio
IgG1: Immunoglobulin-G1
JAG-1: Jagged ½
MRI: Magnetic resonance imaging
NICD: Notch intracellular domain
NSAIDs: Non-steroidal anti-inflammatory drugs
ORR: Objective response rate
PDGF: Platelet-derived growth factor
PFS: Progression-free survival
PR: Partial response
QD: Latin abbreviation for “once a day”
RECIST: Response evaluation criteria in solid tumors
RFA: Radiofrequency ablation
RP2D: Recommended phase 2 dose
SARC: Sarcoma Alliance for Research through Collaboration
SD: Stable disease
TBL-1: Transducing β-like protein-1
TBLR-1: Transducing β-like related protein-1
TCF/LEF: T-cell factor/lymphoid enhancer factor
TKI: Tyrosine kinase inhibitor
TRAE: Treatment-related adverse event
USFDA: United States Food and Drug Administration
VEGF: Vascular endothelial growth factor
